# Bibliometric Analysis of Moxibustion Research Trends Over the Past 20 Years

**DOI:** 10.3390/jcm9051254

**Published:** 2020-04-26

**Authors:** Hyejin Park, In-Seon Lee, Hyangsook Lee, Younbyoung Chae

**Affiliations:** 1 Acupuncture & Meridian Science Research Center, College of Korean Medicine, Kyung Hee University, Seoul 02447, Korea; hyejinpark46@khu.ac.kr (H.P.); islee4u@gmail.com (I.-S.L.); erc633@khu.ac.kr (H.L.); 2 Korean Medicine Convergence Research Information Center, Kyung Hee University, Seoul 02447, Korea

**Keywords:** moxibustion, bibliometric analysis, network analysis, total link strength

## Abstract

Objectives: A bibliometric approach using network analysis was applied to identify the development and research trends for moxibustion. This study also examined the network hub of moxibustion research by investigating the collaborative work of organizations and authors. Methods: Academic articles on moxibustion research published from 2000 to 2019 were retrieved from the Web of Science database. Extracted records were analyzed according to publication year, research area, journal title, country, organization, and authors. The VOSviewer program was utilized to visualize the trends in moxibustion research and to explore the influential organizations and authors. Results: Analyses of 1146 original and review articles written in English demonstrated that the number of publications related to moxibustion research has increased consistently over the last 20 years. China issued the most articles in this field, and the most represented research area was integrative complementary medicine. A network analysis based on the co-occurrence and publication year of keywords identified the relevant characteristics and trends of moxibustion research. By assessing the total link strength of organizations and authors, influential organizations and authors who have contributed to moxibustion research were identified. Conclusions: The current study examined research on moxibustion using bibliometric analysis and identified a time-based development of moxibustion research and a global network hub of moxibustion research.

## 1. Introduction

Moxibustion, a typical therapeutic tool of traditional East Asian medicine, is one of the most widely used treatment methodologies in the current healthcare system of East Asia. Moxibustion has been used to prevent and treat diseases for more than 2500 years in traditional Chinese medicine [1], and 67% of Korean medical doctors were reported to use moxibustion alone or in combination with their standard clinical practices [2]. Moxibustion uses thermal and chemical stimulants by burning herbal materials [3], and the combination of heat (burning pain and heat stress), tar (extracts), aroma (fumes), and psychological stress are presumed to act as therapeutic agents in moxibustion [4]. Its efficacy is mainly focused on warming and nourishing, indicating that heat stimulation and the chemical action of the ignited moxa are the most crucial variables in moxibustion [1].

Previous research has investigated the effectiveness and mechanisms of moxibustion for specific medical conditions [5]. Clinical trials have demonstrated the efficacy of moxibustion for treating suboptimal health status in pre-menopausal and post-menopausal women [6] and the possibility of using moxibustion to promote cephalic version in breech presentations [7]. Several systemic reviews showed that clinical applications of moxibustion were useful as an adjunct to standard care during stroke rehabilitation [8], and demonstrated that moxibustion might be beneficial in the management of irritable bowel syndrome [9]. Furthermore, basic research studies have investigated the underlying mechanisms of moxibustion on specific diseases such as primary dysmenorrhea [10] and post-inflammatory irritable bowel syndrome [11]. Considering the increasing number of published studies and the diversity of topics, a quantitative analysis of studies on moxibustion is needed to understand the current status of moxibustion research.

Bibliometrics is a qualitative and quantitative analysis method used to assess the relationships and impacts of publications, journals, countries, and researchers within a certain research field using mathematical and statistical tools [12]. Bibliometrics can be utilized to provide overviews of large volumes of academic articles and systematically estimate the patterns of research trends. Moreover, it enables identification of the influential publications, authors, journals, organizations, and countries in a specific research area [13], and these analyses can provide data to inform policy making and clinical guidelines in the future [14]. Bibliometric analyses have been employed to assess research trends in a variety of fields, including complementary and alternative medicines. For example, bibliometric studies have been used to evaluate acupuncture research over the past 20 years [15,16] and to investigate the overall trends in acupuncture clinical trials [17]. However, no studies have applied bibliometric analyses to moxibustion research.

The present study used bibliometrics to explore the current status of research on moxibustion over the past 20 years based on a network analysis examining the patterns of moxibustion research.

## 2. Methods

### 2.1. Data Sources and Search Strategy

All data were retrieved from the Web of Science via the Kyung Hee University Library website on 5 February 2020, using the following search terms: (moxibustion OR moxa). The Web of Science provides comprehensive publication data and is widely accepted and frequently used for the analysis of scientific publications. The publication period considered in this study was from 1 January 2000 to 31 December 2019, and a total of 1270 articles were identified. Articles that were not original articles or reviews (*n* = 95) and those that were not written in English were excluded (*n* = 29); thus, 1146 articles written in English were ultimately included in the final analyses.

### 2.2. Data Acquisition and Cleaning

All data from the Web of Science were imported into VOSviewer v.1.6.11 (Centre for Science and Technology Studies, Leiden University, Leiden, The Netherlands), which is commonly used to analyze and visualize relationships among authors, countries, co-citations, and the terms used in the articles. Publications were sorted and systematically assessed according to publication year, research area, journal title, countries, organizational affiliations, and authors. Additionally, the frequencies of keywords extracted from the articles were included in the network analysis.

Before the data were analyzed by VOSviewer, duplicated terms were removed. Some keywords with synonyms, such as (“rats” and “rat”) and (“moxibustion” and “moxa”), were merged into one word. The same process was performed for author names. Cleaned and organized data were then imported into VOSviewer.

### 2.3. Data Analysis

The visualization of similarities (VOS) mapping method was used to estimate similarity (affinity) according to association strength, where higher association strength refers to a greater similarity between terms, and a larger number of publications in which two items co-occur indicates that the terms are more closely similar to each other. The link strength between two nodes denotes the frequency of co-occurrence. It can be used as a quantitative index to describe the relationship between two nodes [18]. In the case of co-occurrence analysis, the link strength between keywords displays the number of publications in which keywords occur together. Keywords represent the article research themes, so co-occurring keywords reveal associations in the underlying themes among publications. Keywords were defined as words used more than 20 times in titles and abstracts across all publications, and co-words were defined as words that co-occurred in the article titles and abstracts. By logging the keywords based on the average publication year, it was possible to identify both popular topics and research trends in moxibustion research.

The total link strength of a node is the sum of the link strengths of this node over all the other nodes [19]. In the case of co-authorship analysis, the link strength between organizations demonstrates the number of publications co-authored by two affiliated organizations. In contrast, the total link strength indicates the total strength of the co-authorship links of a given organization with other organizations [20].

In VOSviewer, each node, keyword, or organization is represented by a circle, and the diameter and label size of the circle indicate the number of occurrences. The label size of a term denotes the number of publications, and the distance between two terms denotes the degree of association. Linked terms are automatically grouped and clustered using different colors. The number of clusters can vary depending on the threshold of similarity between the nodes. The resolution for clustering was adjusted to the proper level as required.

## 3. Results

### 3.1. Global Trends in Studies Investigating Moxibustion Over the Past 20 Years

**Figure 1:**
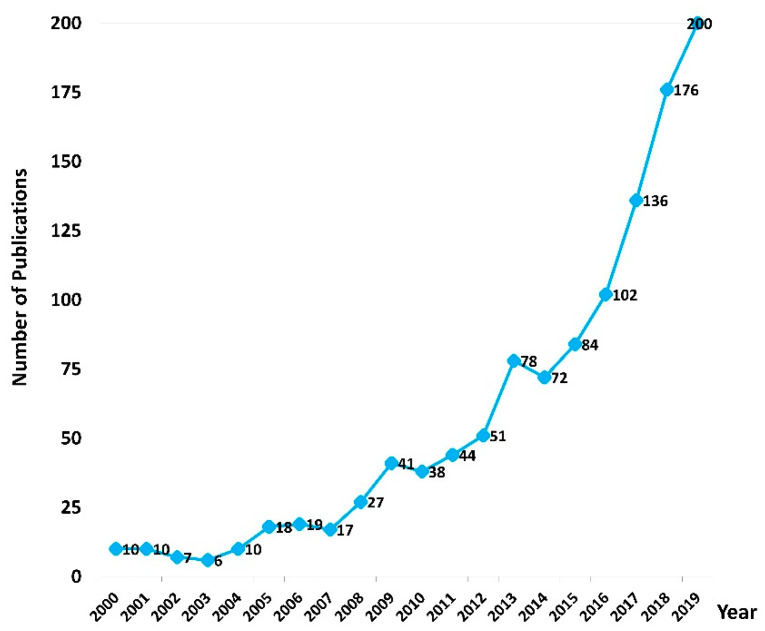
Number of publications investigating moxibustion by year over the past 20 years.

The number of articles related to moxibustion research has consistently increased over the past 20 years, but with some variations among years (Figure 1). Among the 21 countries/regions identified in the current study, China published the most articles (62.7%), followed by South Korea (12.8%) and the United States (10.7%; Table 1).

### 3.2. Analysis of Research Areas and Journals

Of the 61 research areas that were verified in the present study, the most highly represented research area as judged by the number of articles was integrative complementary medicine (58.6% of all articles), followed by general internal medicine (7.5%), neuroscience and neurology (6.2%), research experimental medicine (5.1%), and cell biology (3.8%; Table 2).

Of the 253 journals identified in this study, the World Journal of Acupuncture–Moxibustion issued the most articles (14.9%), followed by Evidence-Based Complementary and Alternative Medicine (10.7%), and Journal of Acupuncture and Tuina Science (5.8%; Table 2).

### 3.3. Analysis of Keywords

**Figure 2:**
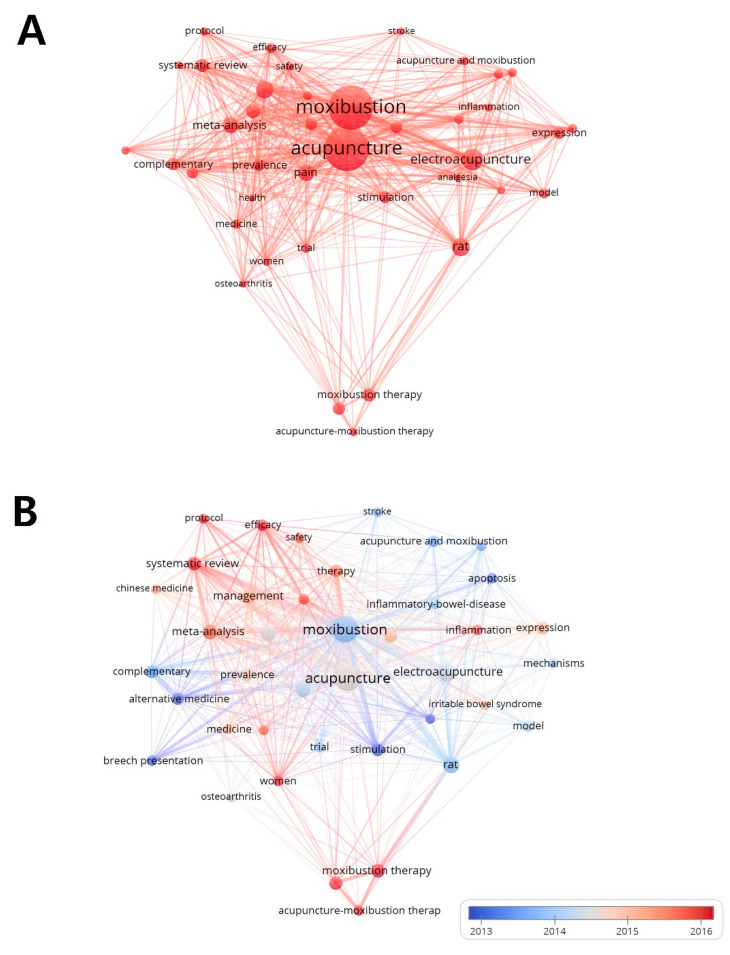
Analysis of the keywords that occurred more than 20 times in the title and abstract fields. (**A**) Mapping of the keywords of studies pertaining to moxibustion. (**B**) Distribution of keywords according to the average publication year (blue: earlier; red: later).

Keywords from the 1146 publications assessed in the present study were analyzed using VOSviewer (Figure 2A). Forty keywords occurred more than 20 times in the title and abstract fields across all the articles. Table 3 shows the occurrences, average publication year, and average citation numbers of the 40 keywords. The top three keywords according to the weight of occurrences were moxibustion (346 occurrences), acupuncture (343), and electro-acupuncture (110), suggesting that moxibustion has been studied along with different treatment modalities, such as acupuncture or electro-acupuncture.

The characteristics of moxibustion research were examined by analyzing these 40 keywords, with moxibustion studies divided into two categories: basic studies, with topics such as rat (82 occurrences), stimulation (47), expression (44); and clinical studies, with keywords including randomized controlled trial (80 occurrences), meta-analysis (65), and systematic review (52). Independent of the type, research on the application of moxibustion was conducted for particular medical conditions such as inflammatory bowel disease (29 occurrences), breech presentation (25), inflammation (25), irritable bowel syndrome (24), osteoarthritis (20), and stroke (20).

The keywords were color-coded by VOSviewer based on the average publication year as shown in Figure 2B, where blue represents relatively early and red represents later average publication years. The most recent keywords were “acupuncture–moxibustion therapy” (average publication year: 2018.0), “moxibustion therapy” (2017.7), “protocol” (2017.3), “acupuncture therapy” (2017.2), and “women” (2016.4). In contrast, the most common keywords in earlier moxibustion studies were “analgesia” (average publication year: 2011.6), “breech presentation” (2011.8), “stimulation” (2012.5), “alternative medicine” (2013.1), and “apoptosis” (2013.2).

### 3.4. Analysis of Main Diseases Using Moxibustions

**Figure 3:**
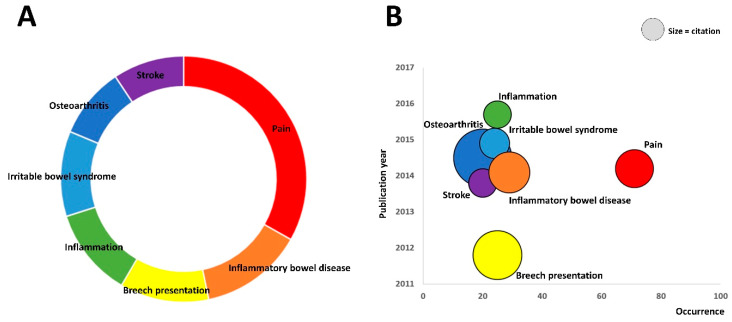
Main diseases using moxibustions using keywords analysis. (**A**) The seven main diseases using moxibustions were found among 40 keywords through keywords analysis: pain (33.2%), inflammatory bowel disease (13.6%), breech presentation (11.7%), inflammation (11.7%), irritable bowel syndrome (11.2%), osteoarthritis (9.3%), and stroke (9.3%). (**B**) When we considered the research trends by average publication year, breech presentation was the oldest (in yellow) and inflammation was the most recent (in green). When we extracted the impact of research by average citation number, osteoarthritis had the highest citation number (in blue) and inflammation had the lowest citation number (in green). The size of the circle represents the average citation number of each keyword.

The seven main diseases using moxibustions were found among 40 keywords through keywords analysis: pain (33.2%), inflammatory bowel disease (13.6%), breech presentation (11.7%), inflammation (11.7%), irritable bowel syndrome (11.2%), osteoarthritis (9.3%), and stroke (9.3%; Figure 3A). When we considered the research trends by average publication year, breech presentation was the oldest (average publication year: 2011.8) and inflammation was the most recent (2015.7). When we extracted the impact of research by average citation number, osteoarthritis had the highest citation number (average citation number: 23.9) and inflammation had the lowest citation number (5.6; Figure 3B).

### 3.5. Analysis of Organizations and Authors

**Figure 4:**
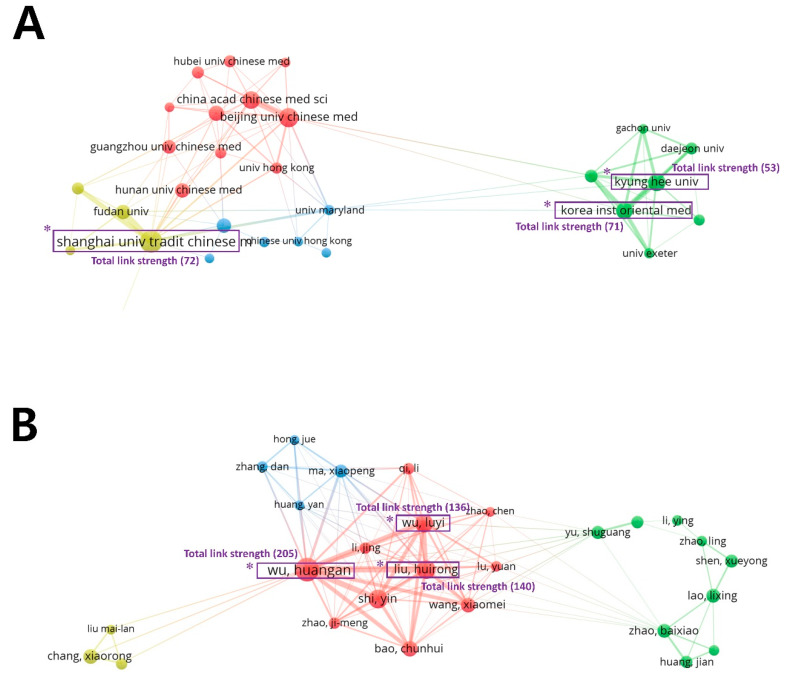
Network map of organizations and authors. (**A**) Distribution of 29 organizations that had at least 10 publications, according to the number of documents. These were classified into four clusters. (**B**) Distribution of 27 authors who had at least eight publications according to the number of documents. These were classified into four clusters. * Represents the top three organizations and the authors with the highest total link strengths.

Table 4 shows the top 10 organizations represented in the 1146 articles identified in the present study. Shanghai University of Traditional Chinese Medicine issued the most articles (9.9%), followed by the Beijing University of Chinese Medicine (5.9%). The organizational affiliations of the publications were analyzed using VOSviewer (Figure 4A), and 29 organizations were found to have published more than 10 articles each (total of 1144). These 29 organizations were classified into four clusters: Cluster 1, with 11 organizations, including Beijing University of Chinese Medicine (410 citations), China Academy of Chinese Medical Sciences (211 citations), and Chengdu University of Traditional Chinese Medicine (161 citations); Cluster 2, with seven organizations, including Kyung Hee University (646 citations), Korea Institute of Oriental Medicine (527 citations), and The University of Exeter (347 citations); Cluster 3, with six organizations, including Harvard University (337 citations), The University of Maryland (325 citations), and China Medical University (183 citations); and Cluster 4, with four organizations, including Shanghai University of Traditional Chinese Medicine (813 citations) and Fudan University (331 citations).

Table 4 shows the top 10 most frequently identified authors based on the author lists for the articles included in the present study. Huangan Wu of Shanghai University of Traditional Chinese Medicine authored or co-authored the most articles (61 articles), followed by Huirong Liu of Shanghai University of Traditional Chinese Medicine (40 articles) and Luyi Wu of the Shanghai University of Traditional Chinese Medicine (36 articles). Authors of the 1146 publications were analyzed using VOSviewer (Figure 4B). The results showed that 27 of 3739 authors appeared more than eight times each; these authors were classified into four clusters: Cluster 1, with 11 authors, including Huangan Wu (565 citations), Huirong Liu (441), and Yin Shi (314); Cluster 2, with nine authors, including Xueyong Shen (238 citations), Lixing Lao (217), and Ling Zhao (126); Cluster 3, with four authors, including Xiaopeng Ma (92 citations); and Cluster 4, with three authors, including Xiaorong Chang (16 citations).

### 3.6. Analysis of Total Link Strength of Organizations and Authors

The total link strength of a node, which is the sum of link strengths of this node over all the other nodes, functions as a standard for weighing an attribute. Therefore, the organizations and authors with high total link strengths were taken to have leading roles, and they, therefore, formed the network hub of moxibustion research. Table 5 shows the top 10 organizations and authors with the highest total link strength across the 1146 articles identified in the present study. Shanghai University of Traditional Chinese Medicine had the strongest total link strength (total link strength: 72), followed by Korea Institute of Oriental Medicine (71), and Kyung Hee University (53). In terms of the authors, Huangan Wu had the strongest total link strength (total link strength: 205), followed by Huirong Liu (140) and Luyi Wu (136).

## 4. Discussion

Our bibliometric analysis of moxibustion research published over the last two decades showed that the total number of articles per year has increased steadily during this period. The most well-represented research areas based on the number of articles were integrative complementary medicine, general internal medicine, neuroscience and neurology, research experimental medicine, and cell biology. Over half of the moxibustion research articles (58.6%) concerned integrative complementary medicine, and the major journals that published moxibustion research articles were generally related to complementary and alternative medicine.

By analyzing keywords, research trends in a particular field of study and the crucial content of published articles can be identified. In the present study, a network analysis based on the occurrence of keywords revealed the trends in moxibustion research and provided information about the types of studies conducted. We identified two main types of studies: basic studies demonstrating the mechanisms of moxibustion or safety observations, and clinical studies investigating the efficacy of moxibustion for specific medical conditions. The main keywords used in the basic studies type were rat, stimulation, and expression, whereas the main keywords in the clinical studies were randomized controlled trial, meta-analysis, and systematic review. These analyses of keywords showed the overarching trends in moxibustion research.

In the current study, we demonstrated that the seven main diseases using moxibustion treatments were pain, inflammatory bowel disease, breech presentation, inflammation, irritable bowel syndrome, osteoarthritis, and stroke. Most of the diseases were related to neurological disorders or inflammatory diseases. The systematic review articles about moxibustion treatment in stroke rehabilitation and diarrhea-predominant irritable bowel syndrome have shown the clinical effectiveness of the moxibustion treatment and provided the usefulness of moxibustion in various health problems [8,21]. However, systematic reviews and meta-analysis can deal with specific diseases respectively. On the other hand, bibliometric analysis provides the overall map of the usage of moxibustion in diverse diseases and contributes to exploring research trends and identifying new areas for research. When we considered the impact of research by citation number, we found that osteoarthritis had the highest citation number among the seven main diseases referred above. This might be associated with the fact in which many clinical trials were conducted on osteoarthritis with moxibustion treatments in the early periods [22,23]. Analysis of these trends in moxibustion research will find core target diseases of moxibustion treatment and offer the latest insights on the state of the art of this research field.

Additionally, we examined the trends in moxibustion research by analyzing the keywords based on the articles’ average publication year and found that the early studies were focused on analgesia, breech presentation, and apoptosis. The keywords used in more recent articles were acupuncture–moxibustion therapy, moxibustion therapy, acupuncture therapy, and women. In the past, many studies aimed to find evidence to support the use of moxibustion to correct breech presentations [24,25,26]. On the other hand, the keyword “breech presentation” was not found in more recent publications. This might be due to the increased number of articles showing limited evidence to support the application of moxibustion for breech presentation. The keywords with recent average publication years revealed that moxibustion was studied together with acupuncture; for example, a study was conducted to find the effect of the combination of compound laser acupuncture and moxibustion as a treatment for type 2 diabetes mellitus [27]. Research related to the keyword “women” showed that moxibustion was used in various studies to investigate its effectiveness on diseases and symptoms related to women, such as health conditions in the pre- and post-menopausal periods [6] and polycystic ovary syndrome [28]. This suggests that moxibustion therapy is regarded as being effective in women. The research trends in moxibustion studies identified by analyzing the keywords in terms of time were able to provide insights for future subject areas of moxibustion research.

The network analysis of countries, organizations, and authors revealed that many research groups in Asian countries produced high numbers of studies on moxibustion, and the majority of organizational affiliations and authors were in China. In addition to examining the top 10 countries, organizations, and authors behind many academic articles, we identified the influential organizations and authors based on their total link strengths. Half of the top 10 organizations with high total link strength were in South Korea, and four organizations were in China; most of the authors with high total link strengths were also from China. This shows that Chinese researchers have played leading roles in moxibustion research. Among the 10 authors with strong total link strengths, the strongest author, Huangan Wu of Shanghai University of Traditional Chinese Medicine, conducted studies mainly related to modulating gut microbiota and intestinal mucosa immunity using moxibustion stimulation [29,30,31]. Along with Hunagan Wu, other authors such as Huirong Liu and Wu Luyi have also researched similar topics. In the bibliometric analysis, the link strength between two nodes indicates the frequency of co-occurrence, allowing it to be used as a quantitative index to depict the relationship between two nodes [18]. The total link strength of a node is the aggregate of link strengths of this node over all the other nodes. Thus, the total link strength creates an index to help identify the network hub in specific research fields.

This study has some limitations. The Web of Science includes other languages, but we limited the language and only included articles written in English. As other languages made up only about 2.2% of the total articles, our overall findings can be assumed to be roughly the same as results obtained with no language refinement. Furthermore, the number of clusters and the cluster labels in the network analysis can be modulated by changing the clustering resolution according to the author’s subjective viewpoint.

Notably, moxibustion was applied more recently in studies associated with acupuncture, especially in studies investigating the efficacy of combined acupuncture and moxibustion in certain diseases. Considering that moxibustion utilizes acupoints, the localization of moxibustion research is understandable. However, unlike moxibustion, acupuncture has been studied worldwide. One possible reason for this could be concerns about safety issues in moxibustion practice. The safety and effects of moxa smoke have been much debated. Some studies have identified the advantageous effects of moxa smoke, including antibacterial, anti-inflammatory, and anti-aging effects [32,33]. However, other studies have shown possible tumor-specific cytotoxic effects of moxa smoke via its pro-oxidant actions [34,35,36]. Therefore, studies demonstrating the beneficial and hazardous aspects of moxibustion are necessary to understand the effects of long-term and short-term human exposure to moxa smoke and to establish a moxibustion safety verification system [37]. When any lingering questions surrounding the efficacy of moxibustion use are answered by definitive safety analyses, global research on moxibustion will be boosted. Our study results will contribute to supporting research on the use of moxibustion. Moreover, our results will provide insight into the trends in moxibustion research and potential hubs for research in this field.

In conclusion, we showed the extent of moxibustion research over the past two decades by applying various bibliometric analyses. Moxibustion treatments were mainly used to treat pain, inflammatory bowel disease, breech presentation, inflammation, irritable bowel syndrome, osteoarthritis, and stroke. By conducting a network analysis of keywords, authors, organizations, and countries and examining the total link strengths of organizations and authors, we were able to identify global time-based research trends and network hubs of moxibustion research. Our findings will contribute to identify core target diseases of moxibustion and provide various insights for researchers in the future.

## Figures and Tables

**Table 1:** The 10 countries that have published the highest numbers of original and review articles investigating moxibustion.

Rank	Country/Regions	Articles (*n*), % of the Total Number of Articles (1146)
1	China	*n* = 719, 62.7%
2	South Korea	*n* = 147, 12.8%
3	USA	*n* = 123, 10.7%
4	Japan	*n* = 67, 5.8%
5	Germany	*n* = 43, 3.8%
6	England	*n* = 35, 3.1%
7	Taiwan	*n* = 32, 2.8%
8	Australia	*n* = 21, 1.8%
9	Brazil	*n* = 20, 1.7%
10	Canada	*n* = 11, 1.0%

**Table 2:** The top 10 research areas and journals that have produced the highest number of original and review articles investigating moxibustion.

Rank	Research Area	Articles (*n*), % of the Total Number of Articles (1146)	Rank	Journal Title	Articles (*n*), % of the Total Number of Articles (1146)
1	Integrative Complementary Medicine	*n* = 672, 58.6%	1	World Journal of Acupuncture-Moxibustion	*n* = 171, 14.9%
2	General Internal Medicine	*n* = 86, 7.5%	2	Evidence-based Complementary and Alternative Medicine	*n* = 123, 10.7%
3	Neurosciences Neurology	*n* = 71, 6.2%	3	Journal of Acupuncture and Tuina Science	*n* = 66, 5.8%
4	Research Experimental Medicine	*n* = 59, 5.1%	4	Journal of Traditional Chinese Medicine	*n* = 63, 5.5%
5	Cell Biology	*n* = 44, 3.8%	5	Neural Regeneration Research	*n* = 40, 3.5%
6	Gastroenterology Hepatology	*n* = 42, 3.7%	6	Medicine	*n* = 31, 2.7%
7	Engineering	*n* = 28, 2.4%	7	Journal of Alternative and Complementary Medicine	*n* = 30, 2.6%
8	Pharmacology Pharmacy	*n* = 27, 2.4%	8	Acupuncture in Medicine	*n* = 28, 2.4%
9	Geochemistry Geophysics	*n* = 26, 2.3%	9	European Journal of Integrative Medicine	*n* = 27, 2.4%
10	Oncology	*n* = 24, 2.1%	10	Chinese Journal of Integrative Medicine	*n* = 26, 2.3%

**Table 3:** Occurrences, average publication year, and average citations of 40 keywords that have occurred over 20 times in the title or abstract fields across all articles.

	Keywords	Weight (Occurrences)	Score (Avg. Pub. Year)	Score (Avg. Citations)
1	moxibustion	346	2013.9	7.9
2	acupuncture	343	2014.5	8.5
3	electroacupuncture	110	2014.4	6.6
4	rat	82	2013.8	8.0
5	randomized controlled trial	80	2014.5	8.0
6	pain	71	2014.2	10.5
7	meta-analysis	65	2015.6	6.7
8	management	57	2015.1	9.9
9	systematic review	52	2015.9	8.7
10	moxibustion therapy	52	2017.7	0.7
11	prevalence	48	2014.7	8.9
12	complementary	47	2013.5	16.8
13	stimulation	47	2012.5	9.6
14	acupuncture therapy	46	2017.2	1.0
15	traditional Chinese medicine	44	2014.9	9.6
16	expression	44	2014.9	6.1
17	alternative medicine	41	2013.1	21.3
18	therapy	41	2015.6	6.3
19	efficacy	35	2016.0	5.5
20	model	31	2014.2	8.3
21	acupuncture and moxibustion	30	2013.7	7.0
22	inflammatory bowel disease	29	2014.1	12.2
23	trial	28	2013.9	10.6
24	mechanisms	27	2014.2	8.8
25	quality-of-life	27	2015.8	7.9
26	medicine	27	2015.1	7.8
27	apoptosis	27	2013.2	6.5
28	neural regeneration	27	2013.8	5.4
29	acupuncture–moxibustion therapy	26	2018.0	0.1
30	breech presentation	25	2011.8	17.2
31	women	25	2016.4	5.7
32	inflammation	25	2015.7	5.6
33	irritable bowel syndrome	24	2014.9	6.7
34	protocol	24	2017.3	1.8
35	safety	22	2015.6	17.9
36	Chinese medicine	22	2015.1	6.7
37	osteoarthritis	20	2014.5	23.9
38	analgesia	20	2011.6	17.5
39	health	20	2015.7	8.0
40	stroke	20	2013.8	6.0

**Table 4:** The top 10 organizations and authors producing the highest number of original and review articles investigating moxibustion.

Rank	Organization	Articles (*n*), % of the Total Number of Articles (1146)	Rank	Author	Articles (*n*), % of the Total Number of Articles (1146)
1	Shanghai University of Traditional Chinese Medicine	*n* = 113, 9.9%	1	Wu, Huangan	*n* = 61, 5.3%
2	Beijing University of Chinese Medicine	*n* = 68, 5.9%	2	Liu, Huirong	*n* = 40, 3.5%
3	China Academy of Chinese Medical Sciences	*n* = 58, 5.1%	3	Wu, Luyi	*n* = 36, 3.1%
4	Kyung Hee University	*n* = 57, 5.0%	4	Shi, Yin	*n* = 35, 3.1%
5	Korea Institute of Oriental Medicine	*n* = 51, 4.5%	5	Lee, Myeong Soo	*n* = 33, 2.9%
6	Chengdu University of Traditional Chinese Medicine	*n* = 36, 3.1%	6	Chen, Rixin	*n* = 25, 2.2%
7	Nanjing University of Chinese Medicine	*n* = 34, 3.0%	7	Bao, Chunhui	*n* = 24, 2.1%
8	Hunan University of Chinese Medicine	*n* = 32, 2.8%	8	Chang, Xiaorong	*n* = 24, 2.1%
9	Fudan University	*n* = 31, 2.7%	9	Zhao, Baixiao	*n* = 24, 2.1%
10	Guangzhou University of Chinese Medicine	*n* = 27, 2.4%	10	Wang, Jing	*n* = 21, 1.8%

**Table 5:** The top 10 total link strengths of organizations and authors.

Rank	Organization	Total Link Strength	Rank	Author	Total Link Strength
1	Shanghai University of Traditional Chinese Medicine	72	1	Wu, Huangan	205
2	Korea Institute of Oriental Medicine	71	2	Liu, Huirong	140
3	Kyung Hee University	53	3	Wu, Luyi	136
4	Beijing University of Chinese Medicine	49	4	Shi, Yin	112
5	Pusan National University	41	5	Bao, Chunhui	83
6	China Academy of Chinese Medical Sciences	39	6	Wang, Xiaomei	71
7	Fudan University	32	7	Ma, Xiaopeng	58
8	Daejeon University	27	8	Lu, Yuan	36
9	Gachon University	26	9	Huang, Yan	29
10	University of Exeter	23	10	Zhao, Chen	28
